# How Can Servant Leadership Promote Employees’ Voice Behavior? A Moderated Chain Mediation Model

**DOI:** 10.3389/fpsyg.2022.938983

**Published:** 2022-07-04

**Authors:** Hao Chen, Liang Wang, Jingya Li

**Affiliations:** ^1^School of Economics and Management, Wuhan University, Wuhan, China; ^2^Chinese Graduate School, Panyapiwat Institute of Management, Pak Kret, Thailand; ^3^School of Economics and Management, Ningxia University, Yinchuan, China

**Keywords:** servant leadership, psychological security, error learning ability, employees’ voice behavior, work autonomy, learning

## Abstract

**Purpose:**

Based on Social Exchange Theory, this paper constructs and evaluates the mediation model of servant leadership on employees’ voice behavior through psychological security and error learning ability and discusses the moderation role of work autonomy in the model.

**Design:**

This research used 424 employees and their direct superiors as the research objects and then conducted a paired survey at three points. Mplus7.4 software analyzed the empirical data.

**Findings:**

The results are shown servant leadership is positively correlated with employees’ voice behavior, but psychological security and error learning ability play a partial mediation role in the positive impact of servant leadership on employees’ voice behavior. while psychological security and error learning ability play a chain mediation role in the positive impact of servant leadership on employees’ voice behavior. Besides, work autonomy moderates the chain mediation path by enhancing the positive impact of servant leadership on psychological security. the higher the employees’ work autonomy is, the stronger the positive impact of servant leadership on psychological security will be, and the stronger the chain mediation effect of psychological security and error learning ability between servant leadership and employees’ voice behavior.

**Practical Implications:**

Managers should abandon the traditional “command and control” management mode, focus on the servant leadership style and improve employees’ psychological security through a comfortable and independent working environment. Besides, managers should set up a suitable error learning atmosphere mechanism, appropriately allow employees to work, give them a specific work autonomy, increase employees’ work flexibility, and encourage employees to provide a cheerful voice for the development and progress of the organization.

**Origin/value:**

From the perspective of Social Exchange, this study more comprehensively discusses the internal action path of the impact of servant leadership on employees’ voice behavior, enriches the antecedent variables of employees’ voice behavior, expands the existing research on the mediation mechanism of servant leadership, deepens the understanding of the efficiency mechanism of servant leadership, and has enlightenment significance for managers to stimulate employees’ voice better.

## Introduction

In today’s era of the knowledge economy, the organization’s dynamic and complex external environment is becoming increasingly intense. Enterprise development depends not only on the personal wisdom of leaders but also on the wisdom of employees. Human resources become an essential part of enterprises’ survival and sustainable competitive advantage. As the most critical human capital of an enterprise, the value of employees is not limited to work performance and labor ability but also reflected in their ability to actively take part in management decision-making and put forward more effective ideas and suggestions for the organization. Earlier studies have shown that voice behavior is a kind of out-of-role interpersonal communication behavior (Le Pine and Van Dyne, 1998; Bashshur and Burak, 2014). It plays a positive role in organizational reform and innovation and enhances competitiveness (Michael et al., 2014; Chen and Hou, 2016; Burris et al., 2017; Chamberlin et al., 2017). In the research of antecedent variables of voice behavior, leadership style has attracted the attention of scholars (Takeuchi et al., 2012; Allen et al., 2013; Frazier and Bowler, 2015; Yan and Xiao, 2016). Therefore, how to make the employees dare to speak up when they find the problems of the enterprise instead of choosing to avoid and keep silent has become the focus of the academic circles.

Servant leadership is a flexible leadership style guided by integrity, selflessness, and altruism (Smith et al., 2004). It is self-positioned as a service provider, emphasizes soothing employees’ emotions, authorizes employees, and helps employees grow (Ehrhart, 2004; Liden et al., 2008). In leadership, it provides continuous service to employees. It has a strong thinking ability and willingness to sacrifice its interests for the organization and employees (Barbuto and Wheeler, 2006. By reviewing the earlier literature, we have found that servant leadership can effectively affect employees’ psychological state and work behavior (Neubert et al., 2008; Cerit, 2009; Hunter et al., 2013; Wu et al., 2013; Carter and Baghurst, 2014). However, the research on the influence of servant leadership on employees’ voice behavior is minimal. Thus, this study will explore the impact mechanism of servant leadership on employees’ voice behavior from the perspective of subordinates’ feelings.

This study further focuses on the relationship between servant leadership and employees’ voice behavior based on Social Exchange Theory. First, the mean doctrine profoundly influenced most employees in the Chinese context, they are cautious in their words and deeds at work, and their willingness to voice is low (Zhu et al., 2014). Servant leadership prioritizes the employees’ interests, pays attention to the employees’ growth, and gives respect and authorization to employees (Van Dierendonck, 2010. Servant leadership may improve employees’ voice behavior. Second, psychological security is an individual’s psychological feeling and inner state; higher psychological security can motivate employees to be more active at work (Kahn, 1990). As an action orientation, error learning ability can effectively improve the effect of individual reflection and learning from errors (Yu, 2008). Thus, this study will explore the mediation role of psychological security and error learning ability in servant leadership and employees’ voice behavior and explore the mechanism between servant leadership and employees’ voice behavior by constructing a chain mediation path.

**FIGURE 1:**
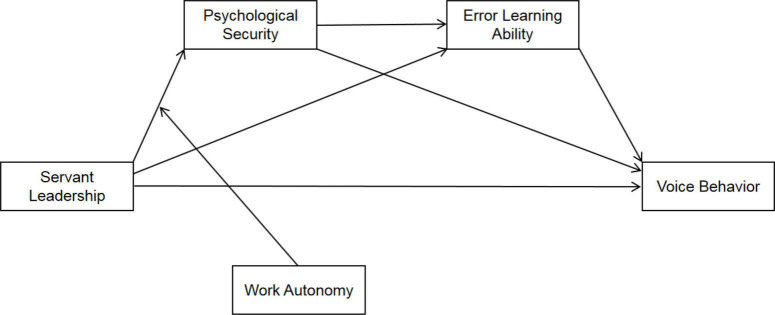
Theoretical hypothesis model.

Employees will inevitably be “overcautious” and “cowardly” at work; as a loose and free working situation in the workplace work autonomy can effectively ease these kinds of employees’ work attitudes and behavior (Sappleton and Lourenco, 2016; Nesheim et al., 2017). Therefore, work autonomy may be an essential boundary condition in servant leadership, affecting psychological security and error learning ability. Based on the points above, from the perspective of Social Exchange Theory, this study aims to build a mediation model for servant leadership to affect employees’ voice behavior through psychological security and error learning ability and explore the moderation role of work autonomy in the model. Practically, this study aims to supply the directional basis for improving employees’ positive voice and problem-solving initiatives for the organization, thus improving the overall operational efficiency. The research model is shown in Figure 1.

## Theoretical Review and Research Hypothesis

### Servant Leadership and Employees’ Voice Behavior

Employees’ voice behavior is a kind of cheerful and spontaneous Out-of-role behavior (Van Dyne et al., 2008) provides constructive suggestions for the organization through interpersonal communication (Le Pine and Van Dyne, 2001; Morrison, 2011). It can effectively improve the operational efficiency of the organization, improve the current situation of the organizational environment, enhance the external adaptability of the organization, and improve the quality of decision-making of leaders, which is an essential basis for the sustainable development, reform, and innovation of the organization (Le Pine and Van Dyne, 1998. From the earlier studies, it has been found that leadership style is an essential situational factor (Morrison, 2014). An upbeat leadership style can effectively promote employees’ voice behavior (Zohar and Luria, 2010; Xu et al., 2015; Svendsen and Joensson, 2016).

As an employee-oriented leader, a servant leader emphasizes that he/she is a server (Russell, 2001) and focuses on the interests and needs of employees (Greenleaf et al., 2002). Servant leaders will delegate, prioritize subordinates, supply emotional comfort, conceptualize skills, help subordinates grow and succeed, behave ethically, create value for the community (Liden et al., 2008), and motivate employees to contribute to the organization (Liden et al., 2015). Generally, servant leaders have the attitude of serving others, helping employees grow and develop, giving employees the right to work independently, and caring for and respecting employees’ benefits and values (Washington et al., 2006). Previous studies have shown that servant leadership can significantly affect individual-level changes in employees’ psychology, work attitude, and behavior (Walumbwa et al., 2010; Chughtai, 2017; Lapointe and Vandenberghe, 2018).

Social Exchange Theory holds that both sides of social exchange supply valuable things to the other side, and the beneficiary will try to repay until the exchange is balanced (Blau, 1964). On the one hand, servant leadership focuses on serving employees. The leadership characteristics of care and respect will make employees feel valued, which will improve their job satisfaction. Employees will reward the organization with more out-of-role behaviors. Servant leadership puts the needs of employees first place, aids and encourages employees in their work, and can provide particular help to employees who are facing difficulties. When employees perceive the genuine care and support of servant leaders, they will actively repay the leaders by working hard. Besides, the servant leadership style is easier to mobilize employees’ enthusiasm and sense of responsibility (Fritz and Sonnentag, 2009) and encourage them to show more work behaviors beneficial to the organization at work to make their contributions to the development of the organization (Bande et al., 2016). Based on the analysis above, the following hypothesis is proposed:

Hypothesis 1 (H1): Servant leadership positively correlates with employees’ voice behavior.

### The Mediation Role of Psychological Security

Psychological security is a characteristic of an individual’s psychological perception and inner state (Kahn, 1990. When employees perceive the risks and uncertainties in the work environment, they will change their psychological state (Bos and Lind, 2002), affecting their cognition and behavior. Research shows that leadership style is one of the critical factors affecting psychological security (Carnelian and Hoffer, 2009). with high psychological security, employees will not be too hesitant in work because of the possible negative impact or adverse consequences on themselves. They can devote themselves to work and give full play to express their true self (Edmondson, 1999, 2004). On the contrary, employees may shrink back with low psychological security because they will cause unnecessary consequences or injuries to themselves, showing “hands tied” behavior.

According to Social Exchange Theory, after receiving a favor from others, the beneficiary will repay somehow. The beneficiary will also have an emotional state of gratitude (McCullough et al., 2001; Tsang and Martin, 2019). On the one hand, employees’ psychological security depends on leaders’ leadership behavior (Edmondson, 1999). In the dynamic and complex organizational environment, servant leadership can keenly detect employees’ concerns about the possible adverse consequences of voice (Xiaoming and Rui, 2015). It eliminates or eases the concerns brought to employees by voice through heartfelt care and comfort. Employees feel a sense of security and it promote employees to speak out their suggestions and ideas more boldly and bravely (Yan and Xiao, 2016).

Servant leadership gives employees the right to complete their work independently. Employees can freely control their time and work style, enabling them to give full play to their talents, control the work progress quickly, and show substantial control, further increasing employees’ sense of security. When employees have high psychological security at work, they will actively find the problems existing in the organization, and actively seek ways to continuously improve the work status, then produce work attitudes and spontaneous behaviors conducive to the organization’s development (George and Brief, 1992. Based on the analysis above, the following hypothesis is proposed:

Hypothesis 2 (H2): Psychological security mediator between servant leadership and employees’ voice behavior.

### The Mediation Role of Error Learning Ability

Error learning ability refers to the individual’s thinking and attribution of the error behavior after he/she has made errors in their work (Frese et al., 2011) and applies accumulated experience and new knowledge learned from errors to decisions or actions (Zhao, 2011; Ju et al., 2015). Error learning ability requires individuals to admit and accept the occurrence and existence of errors (Harteis et al., 2008), which is based on the individual’s correct cognition of errors (Bauer and Mulder, 2007). Error learning ability differs from experiential learning, and individuals will increase observed behavior after errors occur (Dormann and Frese, 1994). It helps individuals explore strategies to solve problems as a precondition for enterprises to innovate and enhance their external adaptability (Starkey, 1998). Individuals with low learning ability usually avoid showing their error behavior (Husted and Michailova, 2002) and even try to cover up or escape responsibility with a fluke mentality.

Earlier studies have shown that error learning ability can lead to positive behavior outcomes; individuals can reflect on their errors and summarize lessons and experiences. It talks about their errors with a cheerful outlook. It puts forward more constructive consequences through practical learning and communication (Pearn et al., 1998) to promote the realization of enterprise goals (Li et al., 2013). Good error learning ability can positively affect employees’ voice tendency. According to Social Exchange Theory, the relationship between people is essentially a social exchange relationship. When individuals are well-treated and supported by others, individuals will give the same positive return (Cropanzano and Mitchell, 2005. Servant leadership can take employees as the center and serve every organization member.

It gradually forms a benign interactive relationship so that employees can perceive leaders’ respect, understanding, and goodwill. They will give back to leaders and organizations with practical actions, and in case of errors, they will not choose to avoid them, and they will not be too cautious at work just to avoid errors. Instead, they can face their errors, regard errors as opportunities to gain experience, and effectively find and reflect on them at work. They gain knowledge and experience from them and actively put forward suggestions and ideas (Van Dyck et al., 2005), which is beneficial for enterprises to form unique competitive advantages (Love and Smith, 2016). Therefore, the following hypothesis is proposed:

Hypothesis 3 (H3): Error learning ability plays a mediator between servant leadership and employees’ voice behavior.

### Chain Mediation Role of Psychological Security and Error Learning Ability

According to the relationship predicted by H1, H2, and H3, this study holds that psychological security and error learning ability further plays a chain mediation role in servant leadership and employees’ voice behavior. The servant leadership style believes that “leaders’ care, respect, and help on employees” can improve employees’ recognition of them and set up mutual trust and respect to feel safe from servant leadership. Even if they make some errors at work, servant leadership treats them fairly. Therefore, employees with an acute sense of psychological security have strong psychological support when making errors at work. They will think more actively, effectively interpret errors and their inducing causes, get knowledge from errors, share knowledge and transfer knowledge to others. In this working atmosphere, employees are more likely to be inspired by their voices. Therefore, the following hypothesis is proposed:

Hypothesis 4(H4): Psychological security and error learning ability play a chain mediation role between servant leadership and employees’ voice behavior. Servant leadership improves employees’ error learning ability by enhancing employees’ psychological security and then affects employees’ voice behavior.

### The Moderation Role of Work Autonomy

Work autonomy is regarded as a working feature (Huber and Glick, 1993), an individual’s freedom, independence, and decision-making power in the working process and working mode (Hackman and Oldham, 1975), and a reflection of an individual’s control overwork. Work autonomy can affect an individual’s work status, thus affecting their work behavior performance (Fuller et al., 2010; Sappleton and Lourenco, 2016). The higher the autonomy of work is, the more complex the individual will work (Liden et al., 2000), thus creating more positive benefits for the organization (Bizzl and Soda, 2011; Nesheim et al., 2017). Specifically, individuals’ feeling of the work environment and their characteristics will affect their psychological state, thus affecting their behavior in the organization. When the work autonomy is high, employees can freely arrange their work tasks and work plans at work. In an organizational environment with work autonomy, it is easier to motivate employees to adopt a positive work (Oldham and Cummings, 1996; Maden-Eyiusta, 2016), thus improving their psychological security. Low work autonomy may lead employees to complete their work in a hostile and passive state, thus reducing their psychological security. Therefore, the following hypothesis is proposed:

Hypothesis 5 (H5): Work autonomy plays a moderation role between servant leadership and psychological security. The higher the employee’s work autonomy is, the stronger the positive relationship between servant leadership and psychological security is.

Moreover, based on Hypothesis 4 and 5, we further infer that work autonomy may moderate the mediation role of psychological security and error learning ability between servant leadership and employees’ voice behavior. When the work autonomy is high, servant leadership will reduce the psychological burden of employees at work, and they will not consider too much psychological burden except work. Psychological security encourages employees to actively respond to errors at work, try to summarize experiences and lessons, and learn from them, so they are likely to break the silence and speak freely about the organization’s interests. Therefore, hypothesis 6 is proposed:

Hypothesis 6(H6): Work autonomy can enhance the positive impact of servant leadership on psychological security and then moderate the chain mediation role of psychological security and error learning ability between servant leadership and employees’ voice behavior.

## Research Design

### Research Samples

This study takes ordinary employees and their direct superiors of several Chinese enterprises as samples and uses offline questionnaires to collect data. To avoid the impact of homology bias, this study uses the method of matching superiors and employees at three separate times to collect data, with an interval of 30 days. The specific investigation process is: The respondents are employees for the first time (T1), and the survey includes the basic information about employees and servant leadership. For the second time (T2), the respondents are employees, and the survey includes psychological security, error learning ability, and work autonomy. For the third time (T3), the respondents are employees’ direct superiors, and the survey includes employees’ voice behavior. Except for some demographic variables, all it scored the questionnaires with Likert 6 points in this study.

We distributed 483 employees’ questionnaires on-site in the first survey, and 474 valid questionnaires were recovered. In the second survey, questionnaires were distributed to the employees who provided valid responses for the first time, and 445 valid ones were recovered. In the third survey, questionnaires were distributed to the direct supervisor of the employees, who provided valid responses for the second time, and 424 valid ones were recovered. The effective recovery rate was 87.8%. In terms of sample structure, most of the employees are male, accounting for 64.7% of the total; in terms of age structure, most of them are young people, and employees under the age of 35 account for 58.2%; in terms of education level, respondents with a bachelor degree or beyond bachelor degree account for 61.1% of the total.

### Variable Measurement

The scales used in this study are mature ones at home and abroad or used by many scholars. Each item adopts the Likert 6-point scale scoring method to measure five main variables: servant leadership, psychological security, error learning ability, voice behavior, and work autonomy. For measuring servant leadership, this study adopts the scale prepared by Liden et al. (2014), with seven items. The representative item is “If I have personal problems, I will ask my leader for help” Cronbach’s α is 0.817. For measuring psychological security, this study adopts the scale compiled by Edmondson (1999), which has seven items. The representative item is “No one will deliberately undermine my efforts” Cronbach’s α is 0.799. For measuring error learning ability, this study adopts the scale developed by Rybowiak et al. (1999), with four items. The representative item is “Errors can provide useful information for me to finish my work” the Cronbach’s α is 0.754. For measuring employees’ voice behavior, this study adopts the scale developed by Schaubroeck et al. (2016), three items. The representative item is “Express new ideas on job-related policies and procedures” Cronbach’s α is 0.760. For measuring work autonomy, this study uses the scale compiled by Liu et al. (2007), three items. The representative item is “I decide how to do the work myself” Cronbach’s α is 0.785.

## Data Analysis and Results

### Confirmatory Factor Analysis

In this study, Mplus 7.4 is used for confirmatory factor analysis of related variables to test the discriminant validity between variables. Results as shown in Table 1, the five-factor model has the best fitting effect (*x*^2^ = 439.117, DF = 199, *X*^2^/DF = 2.207, CFI = 0.928, TLI = 0.916, RMSEA = 0.053, SRMR = 0.046), showing that the five variables in this study have good discriminant validity.

**TABLE 1:** Results of confirmatory factor analysis.

Model	Factor	*x* ^2^	df	*x*^2^/df	CFI	TLI	RMSEA	SRMR
Model1	PS + ELA + SL + WA + VB	1531.349	209	7.327	0.601	0.559	0.122	0.102
Model2	PS + ELA + SL + WA, VB	1460.462	208	7.021	0.622	0.580	0.119	0.101
Model3	PS + ELA + SL, WA, VB	943.861	206	4.582	0.777	0.750	0.092	0.072
Model4	PS + ELA, SL, WA, VB	636.852	203	3.137	0.869	0.851	0.071	0.056
Model5	PS, ELA, SL, WA, VB	439.117	199	2.207	0.928	0.916	0.053	0.046

*N = 424; SL, servant leadership; PS, psychological security; ELA, error learning ability; VB, voice behavior; WA, work autonomy; +:two factors combined as one.*

### Correlation Analysis

The mean value, standard deviation, and correlation coefficient of each variable in this study are shown in Table 2. The data shows that the correlation between variables is consistent with the previous hypothesis of this study: Servant leadership is significantly positively correlated with psychological security (γ = 0.435, *P* < 0.01), error learning ability (γ = 0.498, *P* < 0.01), and voice behavior (γ = 0.365, *P* < 0.01); Psychological security is significantly positively correlated with error learning ability (γ = 0.666, *P* < 0.01), and voice behavior (γ = 0.480, *P* < 0.01);Error learning ability is significantly positively correlated with voice behavior (γ = 0.438, *P* < 0.01).

**TABLE 2:** Mean value, standard deviation, and correlation coefficient of main variables.

	1	2	3	4	5	6	7	8
1. Age	1							
2. Gender	0.106*	1						
3. Education level	–0.001	–0.126**	1					
4. Servant leadership	0.037	0.107*	–0.141**	1				
5. Psychological security	0.086	0.065	–0.128**	0.435**	1			
6. Error learning ability	0.133**	–0.038	–0.114*	0.498**	0.666**	1		
7. Voice behavior	0.129**	0.047	–0.013	0.365**	0.480**	0.438**	1	
8. Work autonomy	–0.115*	–0.129**	0.108*	0.073	0.080	0.083	0.092	1
Mean value (M)	31.55	0.16	2.20	5.184	4.983	5.070	5.103	5.327
Standard deviation (SD)	6.773	0.363	0.528	0.627	0.638	0.615	0.656	0.558

N = 424;**, *stand for P < 0.01, P < 0.05, respectively.

### Hypotheses Test

(1) Test of the significant effect

**FIGURE 2:**
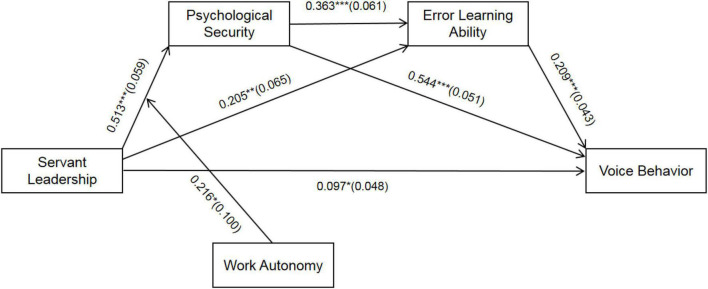
Path coefficient of structural equation. ***, **, *stand for *P* < 0.001, *P* < 0.01, *P* < 0.05, respectively, the coefficient in the figure is the standardization coefficient, and the standard errors are in brackets. The control variables are age, gender, and education level.

Mplus 7.4 tests the structural equation model’s fitting indexes and related assumptions. First, the fitting indexes of the structural equation model are tested. Results are shown in Table 3, the overall fitting degree of the structural equation model is good, and all fitting indices reach an acceptable level. Second, the results of path analysis are shown in Figure 2. Servant leadership positively affects employees’ voice behavior (β = 0.097, *P* < 0.05), so hypothesis H1 is verified.

**TABLE 3:** Fitting indexes of structural equation model.

Fitting index	Critical value (recommended threshold)	Model index	Fit or not
*x* ^2^	the lower, the better	462.783	–
df	the lower, the better	202	–
*x*^2^/df	1<*x*^2^/df<3	2.291	fit
CFI	>0.9	0.921	fit
TLI	>0.9	0.910	fit
RMSEA	<0.08	0.055	fit
SRMR	<0.08	0.058	fit

(2) Test of mediation effect

This study uses bootstrap (repeated sampling 5000 times) to test the chain mediation effect of psychological security and error learning ability. The results are shown in Table 4. The overall indirect effect, the total mediation effect value, is the sum of the mediation effects of the three mediation paths (β = 0.361, *P* < 0.001), and the 95% confidence interval is [0.279, 0.452], excluding 0, the effect is significant. Mediation effect of psychological security (β = 0.279, *P* < 0.001), which is significant, and the 95% confidence interval is [0.006, 0.044], excluding 0, so H2 is verified. Mediation effect of error learning ability (β = 0.043, *P* < 0.01), which is significant, and the 95% confidence interval is [0.017, 0.080], excluding 0. So H3 is verified; the Path coefficient between psychological security and error learning ability (β = 0.363, *P* < 0.001) shows that psychological security significantly and positively affects error learning ability. It means the higher the employees’ psychological security is, the higher the level of error learning ability is. The chain mediation effect of psychological security and error learning ability between servant leadership and employees’ voice behavior is significant (β = 0.039, *P* < 0.01), 95% confidence interval is [0.020, 0.067], excluding 0, so H4 is verified.

**TABLE 4:** Test results of mediating chain effect.

Model path	β	S.E.	*P*	95% confidence interval	
				Lower	Upper
Total	0.458	0.064	0.000	0.334	0.581
Direct	0.097	0.048	0.041	0.003	0.190
Total indirect	0.361	0.044	0.000	0.279	0.452
Path1: SL→PS→VB	0.279	0.038	0.000	0.210	0.361
Path2: SL→ELA→VB	0.043	0.016	0.007	0.017	0.080
Path3: SL→PS→ELA→VB	0.039	0.012	0.001	0.020	0.067

*N = 424; SL, servant leadership; PS, psychological security; ELA, error learning ability; VB, voice behavior.*

(3) Test of the moderation effect

**FIGURE 3:**
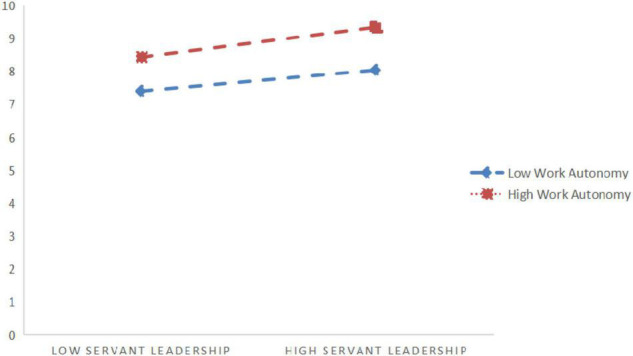
Figure of regulating effect.

It can be concluded from Figure 2 that the interaction between servant leadership and work autonomy has a significant impact on psychological security (β = 0.216, *P* < 0.05), showing that work autonomy significantly moderates the relationship between servant leadership and psychological security. In order to further explain the moderation effect of work autonomy, a simple slope test is carried out according to Aiken et al. suggestions (Aiken and West, 1991), as shown in Figure 3. The results show that when work autonomy is low, the positive impact of servant leadership on psychological security is weak (β = 0.393, *t* = 7.322, *P* < 0.001); when work autonomy is high, the positive impact of servant leadership on psychological security is strong (β = 0.633, *t* = 9.298, *P* < 0.001). With the increase of employees’ work autonomy, the positive impact of servant leadership on psychological security has increased, so H5 is verified.

To test the regulating effect of work autonomy, this study uses Latent Moderate Structural Equations (LMS) to test the moderated chain mediation effect (Fang and Wen, 2018). Results are shown in Table 5. The mediation effect of psychological security and error learning ability between servant leadership and advice behavior is moderated by work autonomy. For employees with high work autonomy (one standard deviation higher than the average value), the indirect effect of servant leadership on voice behavior through psychological security and error learning ability is significantly higher than for employees with low work autonomy (one standard deviation lower than the average). The difference value is 0.018, 95% confidence interval is [0.003, 0.046], excluding 0. Therefore, H6 is verified.

**TABLE 5:** Test results of regulated chain mediating effect.

Regulating variables	Path: SL→PS→ELA→VB			
	Indirect effect	SE	95% confidence interval	
			Lower	Upper
Low WA	0.030	0.003	0.014	0.056
High WA	0.048	0.005	0.024	0.084
Difference	0.018	0.003	0.003	0.046

*N = 424; SL, servant leadership; PS, psychological security; ELA, error learning ability; VB, voice behavior; WA, work autonomy.*

## Discussion and Implication

### Conclusion

Based on Social Exchange Theory, this study discusses the action path of servant leadership affecting employees’ voice behavior through two-source and three-stage data collection. The results show that servant leadership positively correlates with employees’ voice behavior. Psychological security and error learning ability play a separate mediation role and a chain mediation role in the impact of servant leadership on employees’ voice behavior. Work autonomy positively moderates the relationship between servant leadership and psychological security and moderates the chain mediation role of psychological security and error learning ability in servant leadership and employees’ voice behavior.

### Theoretical Significance

(1) From the perspective of Social Exchange, this study explains the impact mechanism of servant leadership on employees’ voice behavior and enriches the research on the antecedent variables of voice behavior. Although previous studies have proved that a critical antecedent variable of voice behavior is leadership style, they are primarily about transformational leadership (Long-Zeng et al., 2011), authentic leadership (Hsiung, 2012), and paternalistic leadership (Chan, 2013) and other leadership styles. As a unique leadership style that is employees-oriented and authorization-oriented, servant leadership can show more commitment and better explain different results (Hoch et al., 2018). However, it is unclear how it affects voice behavior. As a unique leadership style that puts the interests of employees first, servant leadership has low risk and uncertainty, so employees can actively show voice behavior. The research results of this study broaden the research scope of servant leadership and voice behavior and can provide some reference for future related research.

(2) Based on Social Exchange Theory, this study more comprehensively reveals the impact mechanism of servant leadership on employees’ voice behavior. On the one hand, the impact of leadership behavior on employees’ voice behavior can be seen as a psychological mechanism. Servant leadership, an employee’s-centered and democratic participatory leadership style, can eliminate employees’ misgivings and anxiety in completing their work (Tynan, 2005). When employees complete their work with a positive attitude of “master” and “server,” voice behavior will be increased. This study further deepens the understanding of the internal mechanism between servant leadership and employees’ voice behavior. In consideration of “service,” servant leadership gives employees more authorization and support and provides them with a relaxed and accessible working environment. Employees can actively learn about errors and analyze the causes in this working atmosphere. Consequently, their thoughts can be opened more easily, divergent thinking can be generated more quickly, problems can be solved creatively, and then they will voice. Therefore, this study verifies the separate mediation role of psychological security and error learning ability between servant leadership and employees’ voice behavior. This study changes the traditional research ideas and thoroughly uses the chain mediation model to explain the impact mechanism between servant leadership and employees’ voice behavior.

(3) This study verifies the moderation role of work autonomy in the mediation model. As one of the core work characteristics, work autonomy can promote the transformation and regulation of work resources in and out of the role and impact employees’ behaviors (Grant, 2008). Low work autonomy means that employees need to strictly follow the specified procedures, limiting their work initiative and enthusiasm and making them less likely to use resources and spare time to find organizational problems. Therefore, this autonomy is an opportunity and a challenge for employees, which will promote them to open thinking and put forward creative development suggestions at work. Therefore, this study believes that work autonomy can improve the positive effects of servant leadership on employees’ psychological state, action orientation, and work behavior. The empirical results of the moderated chain mediation model constructed by this study make a more comprehensive test of Social Exchange Theory, which helps deepen the academic understanding and development of Social Exchange Theory.

### Management Enlightenment

First, managers should focus on the servant leadership’s behavior style and promote employees’ voices. In Chinese culture, power distance often affects employees, so they become silent, hindering enterprises’ development. On the one hand, managers should break the traditional power distance between superiors and subordinates. It means giving them a lead, paying attention to their manners, maintaining work enthusiasm, and actively communicating with employees about the current enterprise development situation. Leaders should also discuss the internal problems of the enterprises. Managers should focus on serving employees by actively establishing a close working relationship with employees. They should do it by appeasing and caring, providing support and help, respecting employees’ personalities, encouraging employees to constantly challenge themselves, and cultivating employees’ ability to think independently and communicate independently to promote employees’ voices.

Second, managers should improve employees’ psychological security through a comfortable and independent working environment and establish a suitable error learning mechanism. On the one hand, employees with high psychological security think that voice behavior is their internal role, regard voice as a behavior with more advantages than disadvantages, and then like to voice. Therefore, managers should take more measures to enhance employees’ psychological security in daily management practice. It includes flexible working hours and a pleasant working environment to make employees regard the organization as “our home” and will contribute to “our home.” On the other hand, managers should treat employees’ errors rationally, tolerate errors caused by exploration and innovation, and avoid punishing errors caused by short-term goals and laissez-faire. At the same time, managers should consciously provide employees with information that helps them learn and improve. It reduces the panic caused by errors, improves employees’ pressure resistance, makes employees dare to look directly at errors, and breaks the silence, so they dare to voice. Managers should promote employees’ voice behavior to achieve the long-term development goal of the enterprises.

Third, managers should abandon the traditional “command and control” management mode. Instead, appropriately allow employees at work, give them specific work autonomy, and increase employees’ work flexibility. It includes freely arranging work hours, choosing work methods, and self-controlling work progress in promoting employees to practice their ideas at work. Employees create organizational performance and technology with their thoughts, cognition, and emotions. Giving employees the right to choose work methods and work arrangements freely can improve employees’ work enthusiasm and ability and reduce employees’ work constraints. However, it also establishes the style of managers, promotes employees’ follow psychology and loyalty, encourages employees to voice for the development and progress of the organization actively, and constantly explores alternative paths, which will eventually improve organizational performance.

### Research Limitations and Future Prospects

This study also has some limitations, which need to be further explored in the future. First, the samples of this study are mainly servant enterprises. So the conclusions of this study may have some limitations in representativeness. Future researchers can collect data in more industries and obtain more samples to verify the conclusions. Second, this questionnaire uses western scales used for localization research. The behaviors described in these scales may have unique forms in Chinese enterprises, which will affect the research conclusions. Third, this study mainly explores the mediation role of psychological security and error learning ability in the impact of servant leadership on employees’ voice behavior from the individual level. In future research, researchers can try to measure the servant leadership behavior at the team level to verify the effect of behavior under servant leadership. Fourth, the outcome variable of this study is voice behavior. Researchers can further combine other leadership styles to investigate their action mechanisms based on employees’ voice behavior and compare their differences in future research.

## Data Availability Statement

The raw data supporting the conclusions of this article will be made available by the authors, without undue reservation.

## Author Contributions

HC wrote Introduction. LW wrote Discussion. JL contributed to the analysis. All authors contributed to the article and approved the submitted version.

## Conflict of Interest

The authors declare that the research was conducted in the absence of any commercial or financial relationships that could be construed as a potential conflict of interest.

## Publisher’s Note

All claims expressed in this article are solely those of the authors and do not necessarily represent those of their affiliated organizations, or those of the publisher, the editors and the reviewers. Any product that may be evaluated in this article, or claim that may be made by its manufacturer, is not guaranteed or endorsed by the publisher.

## References

[B1] Aiken L. S. West S. G. (1991). *Multiple Regression: Testing and Interpreting Interactions.* Newbury Park, CA: Sage.

[B2] Allen S. L. Smith J. E. Silva N. D. (2013). Leadership style in relation to organizational change and organizational creativity: perceptions from nonprofit organizational members. *Nonprofit Manag. Leadersh.* 24 23–42. 10.1002/nml.21078

[B3] Bande B. Pilar F. F. Concepción V. N. N. Carmen O. N. (2016). Exploring the relationship between servant leadership, intrinsic motivation, and performance in an industrial sales setting. *J. Bus. Ind. Mark.* 31 219–231. 10.1108/JBIM-03-2014-0046

[B4] Barbuto J. E. Wheeler D. W. (2006). Scale development and construct clarification of servantleadership. *Group Organ. Manag. Int. J.* 31 300–326. 10.1177/1059601106287091

[B5] Bashshur M. R. R. Burak O. C. (2014). When voice matters. *J. Manag.* 41 1530–1554. 10.1177/0149206314558302

[B6] Bauer J. Mulder R. H. (2007). Modeling learning from errors in daily work. *Learn. Health Soc. Care* 6 121–133. 10.1111/j.1473-6861.2007.00150.x

[B7] Bizzl L. Soda G. (2011). Self-monitoring, perceived job autonomy, and contextual performance are the paradox of authentic selves and chameleons. *Br.J. Manag.* 22 324–339. 10.1111/j.1467-8551.2011.00747.x

[B8] Blau O. M. (1964). *Exchange and Power in Social Life.* New York, NY: Wiley Press.

[B9] Bos K. V. D. Lind E. A. (2002). Uncertainty management using fairness judgments. *Adv. Exp. Soc. Psychol.* 34 1–60. 10.1016/S0065-2601(02)80003-X

[B10] Burris E. Rockmann K. Kimmons Y. S. (2017). The value of voice (to managers): employee identification and the content of the voice. *Acad. Manag. J.* 60 2099–2125. 10.5465/amj.2014.0320

[B11] Carnelian G. Hoffer J. (2009). High-quality relationships, psychological safety, and learning from failures in work organizations. *J. Organ. Behav.* 30 709–729. 10.1002/job.565

[B12] Carter D. Baghurst T. (2014). The influence of servant leadership on restaurant employee engagement. *J. Bus. Ethics* 124 453–464. 10.1007/s10551-013-1882-0

[B13] Cerit Y. (2009). The effects of servant leadership behaviours of school principals on teachers’ job satisfaction. *Educ. Manag. Adm. Leadersh.* 37 600–623. 10.1177/1741143209339650

[B14] Chamberlin M. Newton D. W. Lepine J. A. (2017). A meta-analysis of voice and its promotive and prohibitive forms: identification of key associations, distinctions, and future research directions. *Pers. Psychol.* 70 11–71. 10.1111/peps.12185

[B15] Chan S. C. (2013). Paternalistic leadership and employee voice: does information share matter? *Hum. Relat.* 67 667–693. 10.1177/0018726713503022

[B16] Chen A. S. Y. Hou Y. H. (2016). The effects of ethical leadership, voice behavior and climates for innovation on creativity: a moderated mediation examination. *Leadersh. Q.* 27 1–13. 10.1016/j.leaqua.2015.10.007

[B17] Chughtai A. A. (2017). Examining the effects of servant leadership on life satisfaction. *Appl. Res. Qual. Life* 13 873–889. 10.1007/s11482-017-9564-1

[B18] Cropanzano R. Mitchell M. S. (2005). Social exchange theory: an interdisciplinary review. *J. Manag.* 31 874–900. 10.1177/0149206305279602

[B19] Dormann T. Frese M. (1994). Error training: replication and the function of exploratory behavior. *Int. J. Hum. Comput. Interact.* 6 365–372. 10.1080/10447319409526101

[B20] Edmondson A. (1999). Psychological safety and learning behavior in work teams. *Adm. Sci. Q.* 44 350–383. 10.2307/2666999

[B21] Edmondson A. C. (2004). “Psychological safety, trust, and learning in organizations: a group-level lens,” in *Trust and Distrust in Organizations: Dilemmas and Approaches*, eds Kramer R. M. Cook K. S. (New York, NY: Russell Sage Foundation), 239–272.

[B22] Ehrhart M. G. (2004). Leadership and procedural justice climate as antecedents of unit-level organizational citizenship behavior. *Pers. Psychol.* 57 61–94. 10.1111/j.1744-6570.2004.tb02484.x

[B23] Fang J. Wen Z. L. (2018). The analyses of moderated mediation effects based on structural equation modeling. *J. Psychol. Sci.* 41 453–458.

[B24] Frazier M. L. Bowler W. M. (2015). Voice climate, supervisor undermining, and work outcomes a group-level examination. *J. Manag.* 41 841–863. 10.1177/0149206311434533

[B25] Frese M. Fay D. Hilburger T. Tag A. (2011). The concept of personal initiative: operationalization. reliability and validity in two German samples. *J. Occup. Organ. Psychol.* 70 139–161. 10.1111/j.2044-8325.1997.tb00639.x

[B26] Fritz C. Sonnentag S. (2009). Antecedents of day-level proactive behavior: a look at job stressors and positive affect during the workday. *J. Manag.* 35 94–111. 10.1177/0149206307308911

[B27] Fuller J. B. Hester K. Cox S. S. (2010). Proactive personality and job performance: exploring job autonomy as a moderator. *J. Manage. Issues* 22 5–51.

[B28] George J. M. Brief A. P. (1992). Feeling hood-doing good: a conceptual analysis of the mood at work-organizational spontaneity relationship. *Psychol. Bull.* 112 310–329. 10.1037/0033-2909.112.2.31 1454897

[B29] Grant A. M. (2008). The significance of task significance: job performance effects, relational mechanisms, and boundary conditions. *J. Appl. Psychol.* 93 108–124. 10.1037/0021-9010.93.1.108 18211139

[B31] Greenleaf R. K. Spears L. C. Covey S. R. (2002). *Servant Leadership: A Journey Into the Nature of Legitimate Power and Greatness.* New York, NY: Paulist Press.

[B32] Hackman J. R. Oldham G. R. (1975). Development of the diagnostic job survey. *J. Appl. Psychol.* 60 159–170. 10.1037/h0076546

[B33] Harteis C. Bauer J. Gruber H. (2008). The culture of learning from mistakes: how employees handle mistakes in everyday work. *Int. J. Educ. Res.* 47 223–231. 10.1016/j.ijer.2008.07.003

[B34] Hoch J. E. Bommer W. H. Dulebohn J. H. Wu D. (2018). Do ethical, authentic, and servant leadership explain variance above and beyond transformational leadership? A meta-analysis. *J. Manag.* 44 501–529. 10.1177/0149206316665461

[B35] Hsiung H. H. (2012). Authentic leadership and employee voice behavior: a multi-level psychological process. *J. Bus. Ethics* 107 349–361. 10.1007/s10551-011-1043-2

[B36] Huber G. P. Glick W. H. (1993). *Organizational Change and Redesign.* New York, NY: Oxford University Press.

[B37] Hunter E. M. Neubert M. J. Perry S. J. (2013). Servant leaders inspire servant followers: antecedents and outcomes for employees and the organization. *Leadersh. Q.* 24 316–331. 10.1016/j.leaqua.2012.12.001

[B38] Husted K. Michailova S. (2002). Diagnosing and fighting knowledge-sharing hostility. *Organ. Dyn.* 31 60–73. 10.1016/S0090-2616(02)00072-4

[B39] Ju X. H. Xie Y. Q. Zhang J. N. (2015). Error learning: a study from the error knowledge flow perspective. *J. Intell.* 34 202–206.

[B40] Kahn W. A. (1990). Psychological conditions of personal engagement and disengagement at work. *Acad. Manag. J.* 33 692–724. 10.5465/256287

[B41] Lapointe E. Vandenberghe C. (2018). Examination of the relationships between servant leadership, organizational commitment, and voice and antisocial behaviors. *J. Bus. Ethics* 148 99–115. 10.1007/s10551-015-3002-9

[B42] Le Pine J. A. Van Dyne L. (1998). Predicting voice behavior in work groups. *J. Appl. Psychol.* 83 853–868.

[B43] Le Pine J. A. Van Dyne L. (2001). Predicting voice behavior in workgroups. *J. Appl. Psychol.* 83 853–868. 10.3389/fpsyg.2021.609953 34712162 PMC8546109

[B44] Li Y. Ma L. Yuan Z. H. Yuan X.-d. (2013). Influence of error management atmosphere on dual innovation the mediating role of knowledge transformation. *Mod. Manag. Sci.* 32 112–114.

[B45] Liden R. C. Wayne S. J. Liao C. Meuser J. D. (2014). Servant leadership and serving culture: influence on individual and unit performance. *Acad. Manag. J.* 57 1434–1452. 10.5465/amj.2013.0034

[B46] Liden R. C. Wayne S. J. Meuser J. D. Hu J. Wu J. Liao C. (2015). Servant leadership: validation of a short form of the sl-28. *Leadersh. Q.* 26 254–269. 10.1016/j.leaqua.2014.12.002

[B47] Liden R. C. Wayne S. J. Sparrowe R. T. (2000). An examination of the mediating role of psychological empowerment on the relations between the job, interpersonal relationships, and work outcomes. *J. Appl. Psychol.* 85 407–416. 10.1037/0021-9010.85.3.407 10900815

[B48] Liden R. C. Wayne S. J. Zhao H. Henderson D. (2008). Servant leadership: development of a multidimensional measure and multilevel assessment. *Leadersh. Q.* 19 161–177. 10.1016/j.leaqua.2008.01.006

[B49] Liu C. Spector P. E. Shi L. (2007). Cross-national job stress: a quantitative and qualitative study. *J. Organ. Behav.* 28 209–239. 10.1002/job.435

[B50] Long-Zeng W. Kun-Peng C. Yuan-Yi C. Gui-Yao G. (2011). Transformational leadership and employee voice behavior: an examination of the mediating mechanisms. *Chin. J. Manag.* 8 61–66.

[B51] Love P. E. D. Smith J. (2016). Toward error management in construction: moving beyond a zero vision. *J. Constr. Eng. Manag.* 142 1–10. 10.1061/(ASCE)CO.1943-7862.0001170 29515898

[B52] Maden-Eyiusta C. (2016). Job resources, engagement, and proactivity: a moderated mediation model. *J. Manage. Psychol.* 31 1234–1250. 10.3390/ijerph19020696 35055518 PMC8775439

[B53] McCullough M. E. Kilpatrick S. D. Emmons R. A. Larson D. B. (2001). Is gratitude a moral effect? *Psychol. Bull.* 127 249–266. 10.1037/0033-2909.127.2.249 11316013

[B54] Michael C. A. Dominey-Howes D. Labbate M. (2014). The antimicrobial resistance crisis: causes, consequences, and management. *Front. Public Health* 2:145. 10.3389/fpubh.2014.00145 25279369 PMC4165128

[B55] Morrison E. W. (2011). Employee voice behavior: integration and directions for future research. *Acad. Manag. Ann.* 5 373–412. 10.5465/19416520.2011.574506

[B56] Morrison E. W. (2014). Employee voice and silence. *Annu. Rev. Organ. Psychol. Organ. Behav.* 1 173–197. 10.1136/bmjqs-2021-014287 35058330

[B57] Nesheim T. Olsen K. M. Sandvik A. M. (2017). Never walk alone: achieving work performance through networking ability and autonomy. *Employee Relat.* 39 240–253. 10.1108/ER-09-2016-0185

[B58] Neubert M. J. Kacmar K. M. Carlson D. S. Chonko L. B. Roberts J. A. (2008). Regulatory focus as a mediator of the influence of initiating structure and servant leadership on employee behavior. *J. Appl.Psychol.* 93 1220–1233. 10.1037/a0012695 19025244

[B59] Oldham G. R. Cummings A. (1996). Employee creativity: personal and contextual factors at work. *Acad. Manag. J.* 39 607–634. 10.5465/256657 256657

[B60] Pearn M. Mulrooney C. Payne T. (1998). *Ending the Blame Culture.* Farnham: Go wer Pub Co.

[B61] Russell R. F. (2001). The role of values in servant leadership. *Leadersh. Organ. Dev. J.* 22 76–83. 10.1108/01437730110382631

[B62] Rybowiak V. Garst H. Frese M. Batinic B. (1999). Error orientation questionnaire (EOQ): reliability, validity, and different language equivalence. *J. Organ. Behav.* 20 527–547. 10.1002/(SICI)1099-1379(199907)20:4<527::AID-JOB886>3.0.CO;2-G

[B63] Sappleton N. Lourenco F. (2016). Work satisfaction of the self-employed: the roles of work autonomy, working hours, gender and sector of self-employment. *Int. J. Entrep. Innov.* 17 89–99. 10.1177/1465750316648574

[B64] Schaubroeck R. D. L. J. M. Lam S. S. K. Peng A. C. (2016). Overcoming the fear factor: how perceptions of supervisor openness led employees to speak up when fearing external threat. *Organ. Behav. Hum. Decis. Process.* 135 10–21. 10.1016/j.obhdp.2016.05.001

[B65] Smith B. N. Montagno R. V. Kuzmenko T. N. (2004). Transformational and servant leadership: content and contextual comparisons. *J. Leadersh. Organ. Stud.* 10 80–92. 10.1177/107179190401000406

[B66] Starkey K. (1998). What can we learn from the learning organization? *Hum. Relat.* 51 531–546. 10.1177/001872679805100405

[B67] Svendsen M. Joensson T. S. (2016). Transformational leadership and change-related voice behavior. *Leadersh. Organ. Dev. J.* 37 357–368. 10.1108/LODJ-07-2014-0124

[B68] Takeuchi R. Chen Z. J. Chenhg S. Y. (2012). Applying uncertainty management theory to employee voice behavior: an integrative investigation. *Pers. Psychol.* 65 283–323. 10.1111/j.1744-6570.2012.01247.x

[B69] Tsang J. A. Martin S. R. (2019). Four experiments on the relational dynamics and prosocial consequences of gratitude. *J. Posit. Psychol.* 14 188–205. 10.1080/17439760.2017.1388435

[B70] Tynan R. (2005). The effects of threat sensitivity and face giving on dyadic psychological and upward communication. *J. Appl. Soc. Psychol.* 35 223–247. 10.1111/j.1559-1816.2005.tb02119.x

[B71] Van Dierendonck D. (2010). Servant leadership: a review and synthesis. *J. Manag.* 37 1228–1261. 10.1186/s12961-022-00820-7 35246139 PMC8894559

[B72] Van Dyck C. Frese M. Baer M. Sonnentag S. (2005). Organizational error management culture and its impact on performance: a two-study replication. *J. Appl. Psychol.* 90 1228–1240. 10.1037/0021-9010.90.6.1228 16316276

[B73] Van Dyne L. Kamdar D. Joireman J. (2008). In-role perceptions buffer the negative impact of low LMX on helping and enhance the positive impact of high LMX on voice. *J. Appl. Psychol.* 93 1195–1207. 10.1037/0021-9010.93.6.1195 19025242

[B74] Walumbwa F. O. Hartnell C. A. Oke A. (2010). Servant leadership, procedural justice climate, service climate, employee attitudes, and organizational citizenship behavior: a cross-level investigation. *J. Appl. Psychol.* 95 517–529. 10.1037/a0018867 20476830

[B75] Washington R. R. Sutton C. D. Field H. S. (2006). Individual differences in servant leadership: the roles of values and personality. *Leadersh. Organ. Dev. J.* 27 700–716. 10.1108/01437730610709309 10108429

[B76] Wu L. Z. Tse C. Y. E. Fu P. Kwan H. K. Liu J. (2013). The impact of servant leadership on hotel employees’ “servant behavior”. *Cornell Hosp. Q.* 54 383–395. 10.1177/1938965513482519

[B77] Xiaoming T. Rui L. (2015). Canself-sacrificial leadership promotes employee proactive behavior? The mediating effect of felt obligation and its boundary conditions. *Acta Psychol. Sin.* 47 1472–1485. 10.3724/SP.J.1041.2015.01472

[B78] Xu A. J. Loi R. Lam L. W. (2015). The bad boss takes it all: how abusive supervision and leader-member exchange interact to influence employee silence. *Leadersh. Q.* 26 763–774. 10.1016/j.leaqua.2015.03.002

[B79] Yan A. M. Xiao Y. G. (2016). Servant leadership and employee voice behavior: a cross-level investigation in China. *Springer Plus* 5:1595. 10.1186/s40064-016-3264-4 27652168 PMC5026985

[B80] Yu F. L. T. (2008). Uncertainty, learning and error elimination: Taiwanese entrepreneurs in Mainland China. *J. Asia Pac. Bus.* 9 248–270.

[B81] Zhao B. (2011). Learning from errors: the role of context, emotion, and personality. *J. Organ. Behav.* 32 435–463. 10.1002/job.696

[B82] Zhu H. Chen X. L. Qian J. X. (2014). Charting the development of social and cultural geography in Mainland China: voices from the inside. *Soc. Cult. Geogr.* 15 255–283. 10.1080/14649365.2014.883423

[B83] Zohar D. Luria G. (2010). Group leaders as gatekeepers: testing safety climate variations across levels of analysis. *Appl. Psychol.* 59 647–673. 10.1111/j.1464-0597.2010.00421.x

